# Application of the MEMS Accelerometer as the Position Sensor in Linear Electrohydraulic Drive

**DOI:** 10.3390/s21041479

**Published:** 2021-02-20

**Authors:** Dominik Rybarczyk

**Affiliations:** Institute of Mechanical Technology, Poznan University of Technology, 60-965 Poznań, Poland; dominik.rybarczyk@put.poznan.pl

**Keywords:** electrohydraulic drives, accelerometers, MEMS, control

## Abstract

Various distance sensors are used as measuring elements for positioning linear electrohydraulic drives. The most common are magnetostrictive transducers or linear variable differential transformer (LVDT) sensors mounted inside the cylinder. The displacement of the actuator’s piston rod is proportional to the change in the value of the current or voltage at the output from the sensor. They are characterized by relatively low measurement noise. The disadvantage of presented sensors is the need to mount them inside the cylinders and the high price. The article presents preliminary research on the replacement of following sensors and the use of a microelectromechanical system (MEMS) accelerometer as a measuring element in the electrohydraulic drive control system. The control consisted of two phases: at first, the signal from the acceleration sensor was analyzed during the actuator movement, based on the value determined from the simplified model implemented on the controller. In the range of motion in which the dynamics were the lowest, the signal was integrated and the obtained value was used in the second phase of motion. In the correction phase, a new set point was determined. Conducting the research required building a dedicated research stand. The author conducted the simulation and experimental research.

## 1. Introduction

Various types of distance-measuring sensors are used for positioning electrohydraulic servo drives. The structure of the electrohydraulic servo drive has been described, among others, in [1,2]. The most common are magnetostrictive transducers or linear variable differential transformer (LVDT) sensors. These sensors are mounted inside the cylinders. They are characterized by low measurement noise and high resolution [3,4]. In the case of magnetostrictive sensors mounted inside actuators, manufacturers indicate parameters such as the linearity value. In case of a wrong reading, it is not aggregated. Their disadvantage is the relatively high price and the need to replace the entire drive unit if it is necessary to introduce position or speed measurements. The solution to this problem may be the use of inertial sensors mounted on selected elements of machines (including pistons). Sensors of this type are often made with micromachined microelectromechanical system (MEMS) technology. An acceleration sensor can theoretically be used to measure linear displacement. Unfortunately, simple double integrations generate large amounts of error resulting from the physics of operating this type of sensor, which is described later in the article. The problem can be partially solved by applying a filter to the signal, e.g., a low-pass filter. Information on how to deal with this problem and the application of inertial sensors in various types of drives is provided in Section 2.

The article presents a novel method of controlling a linear electrohydraulic drive based on data from a MEMS accelerometer. A simplified model of a hydraulic drive was also used in the control algorithm. A detailed description of the method is presented later in the article. This article focuses on the compensation of the supply pressure. This method, however, can be effectively used to compensate for other disturbances, such as the influence of load or a change in operating temperature.

Before starting the development of the control system, the author reviewed the literature, focusing mainly on the use of MEMS accelerometers in hydraulic systems and on determining new position values based on the data read from them. The characteristics of this type of sensor can be found, among others, in the works by Zeimpekis et al. and Li et al. [5,6]. The future of MEMS technology is described in [7].

The method of selecting MEMS sensors for vibration measurement is described in the publication [8]. The performance and suitability for vibration measurement were assessed for two commercially available accelerometers and one present in a smart phone. The authors compared them with a reference conventional piezoelectric accelerometer. They indicated numerous advantages of such systems, such as low cost, miniature in size and wireless communications.

Although MEMS accelerometers constructions have been known for many years, various research groups are still working on the modifications and improvements. The article [9] describes a new type of MEMS accelerometer made from steel and fabricated using a micro-wire electrical discharge machining (μWEDM) process. Using this fabrication method, it is possible to achieve a huge proof mass that has a prominent effect on reducing noise. The proposed construction is also more resistant to damage because of the high yield stress and fracture toughness of the steel. Despite these advantages, this solution is still in the research phase.

In the article [10], the authors described the use of an accelerometer as a part of an inertia sensor to estimate the angle of hydraulic support. Roll and roll angles are estimated with a MEMS gyro and accelerometer, and accuracy is not reliable over time. In order to eliminate the measurement error of the sensors and to obtain a very accurate assessment of the position of the system, a non-trace Kalman filter based on quaternions was used in accordance with the characteristics of the magnetometer, accelerometer and gyroscope complement.

The paper [11] presents an innovative strategy of passive reduction in the source of noise transmitted by fluids, based on indexing the axial piston assembly in tandem with the use of symmetrical lines. Analytical tests were carried out and then confirmed in a simulation test. Experiments on the proposed strategy confirmed its effectiveness at the first and third harmonics of the fundamental pump, and thus, a reduction in pressure ripples was achieved.

The article [12] proposes a damage detection system for a hydraulic axial piston pump. Given the changing operating conditions and the implication of frequent faults, it is difficult to accurately monitor machine faults in the actual operating process using up-to-date fault diagnosis methods. The authors proposed an integrated, intelligent fault diagnosis method based on convolutional neural network (CNN) and continuous wavelet transformation (CWT), combining feature extraction and classification. The results of the experiment are characterized by greater classification accuracy in comparison with other models. It has been shown that the proposed approach is effective and stable in the diagnosis of hydraulic axial piston pump failures.

The problem of using an acceleration sensor when overcoming terrain obstacles for people moving in a wheelchair has been described, among others, by the author of the current text in the publication [13]. In the described system, however, the sensor was not used to measure the change in position in linear motion but to measure the change in the angular position of a wheelchair. A big problem was the wheelchair vibrations, generated mainly by the ground (e.g., when a wheelchair passed over cobblestones). To compensate for them, a complementary filter was used.

The problem of measurement noise and drift (in the case of a gyroscope) is particularly visible in the case of low-cost sensors. Research is being carried out on the construction of more complex systems. The article by Dong [14] describes a system focusing on long-term load stability and thermal drift. Most of them, however, are relatively high-priced components or are prototypes or experimental models.

An overview of the state of knowledge in the field of machine condition monitoring is presented in the article [15]. As the authors point out, it is mainly used in various types of vibration monitoring systems. Data filtration is also a significant problem.

An important issue from the point of view of research with the use of acceleration sensors is to have tools that enable their analysis. The authors of the article [16] created a dedicated application that enabled the simultaneous recording of machining forces, acceleration, noise and/or acoustic pressure (more variables can be added by selecting the appropriate I/O modules).

The article [17] raises a very important issue regarding the way of reading data and their connection with electronic measurement cards. The authors study how the mass and the lightweight connecting cable of an ultra-miniature accelerometer sensor influence the frequency response function (FRF) of thin-wall aluminum workpieces, and its influence over the stability analysis of the milling process. In the performed research, the authors used the method of structural modification and finite element simulations to quantify the mass of the accelerometer and the influence of the connecting cable. A vibrometer was used as the reference system. The authors found that the predicted stability lobes agree well with experimental data if the structural modification method is used to compensate the accelerometer measurements. One solution to this problem may be a significant reduction in the length of the cables connecting individual elements and the introduction of digital communication. In the Bosch BNO055 module [18], used as a position sensor in the presented electrohydraulic drive, a digital inter-integrated circuit (I2C) interface is used for communication. Digital kinds of interfaces reduce the amount of measurement noise generated on transmission lines. The disadvantage of this solution is the fact that many programmable logic controllers (PLCs) do not have an I2C interface. In the present work, this problem was solved by the use of a microcontroller, which was an intermediary system between the MEMS sensor module and the superior control system. The method of connection is described in more detail in Section 5.

Based on the literature review, it can be concluded that a MEMS-type acceleration sensor has not been used so far in the way presented by the author for positioning the electrohydraulic drive. The authors of the above-mentioned papers focused mainly on the use of this type of sensor for diagnostics of various types of hydraulic systems, examining parameters such as vibrations [19,20,21,22]. Other popular applications are measurements of changes in angular position, where the signal from the acceleration sensor is combined with the signal from a gyroscope, among others. The conducted literature review showed problems related to measurements made with accelerometers such as measurement noise and the need to filter the signal. The author thus proposes his own original control algorithm in which an acceleration sensor is used to compensate for disturbances such as supply pressure change in the feedback system.

## 2. Description of the Control Method

One of the disadvantages of MEMS accelerometers is the presence of measurement noise [23]. The authors of the publication [24] described the noise characteristics of MEMS accelerometers at different acceleration values. The experimental results indicated the presence of 1/f noise at low frequencies and Gaussian white noise at high frequencies. The size of the spectral noise density depends on the acceleration of the motion. For this reason, a slightly different approach had to be used in the proposed solution. First, it was not possible to use simple signal double integration. It was necessary to use an additional mechanism working independently. For this purpose, the drive model implemented in the controller was used. The model was controlled by a PID controller. It developed the main control signal, which was then corrected on the basis of signals from the accelerometer.

**Figure 1:**
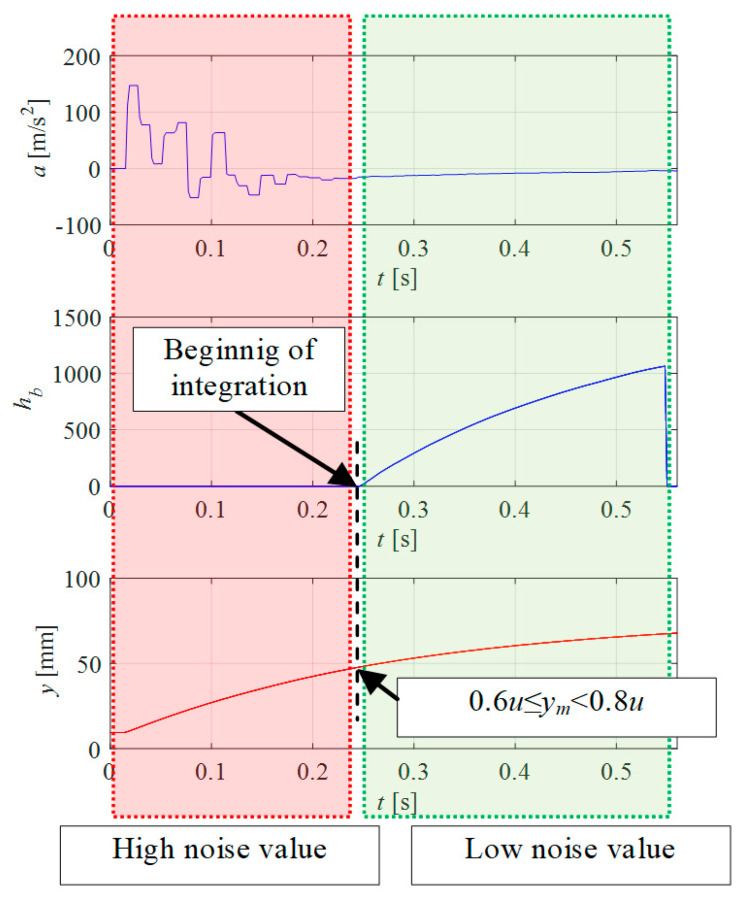
Waveforms of acceleration values and *h_b_* value during changing of the drive position.

Initial research was first carried out to determine how the data from the acceleration sensor could be used. For this purpose, the signal *y_m_* was applied to the valve input, coming only from the previously implemented model. Its description can be found later in the article (Section 3). In the first phase of the movement, the signal amplitude and the noise value are the highest (Figure 1, mark red color). The dynamics of the piston rod movement depend on the signal from the model *y_m_*. In the first phase, the dynamics are greater than in the second phase. When the speed of movement decreases, much smaller oscillations are seen in the sensor signal. The signal from this phase of motion is integrated into the controller according to the following relationship Equation (1):

(1) $$\{\begin{array}{cc} h_{a}=\int a\left(t\right)\;dt & \mathrm{for}\;0\leq y_{m}<0.6u\\ h_{b}=\int a\left(t\right)\;dt & \mathrm{for}\;0.6u\leq y_{m}<0.8u \end{array}$$

where *u*—set-point position; *h_a_*, *h_b_*—integral values of signal from acceleration in different range.

The integral values of the acceleration *h_a_* and *h_b_* in the table come from the analog signal converted into an integer variable in the programmable logic controller (PLC). Table 1 and Table 2 present the selected test results. 

In order to compare the values, the mean deviation *σ*, arithmetic average, standard deviation estimator *s_x_* and measurement uncertainty *u*(*x*) (type A) were determined. The maximum value of mean deviation σ was 23.92 for the *h_b_* parameter and 1469.28 for the *h_a_* parameter. The integral values of the signal *h_b_* are characterized by the few-times smaller and much more similar values compared to the *h_a_* values.

**Figure 2:**
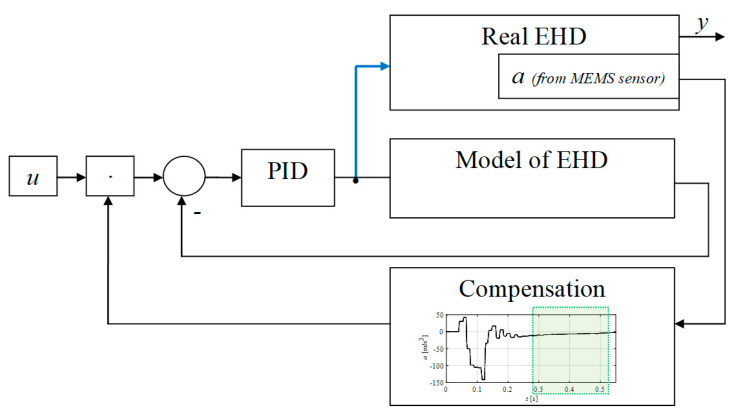
Control system schematic (EHD—electrohydraulic drive).

A schematic diagram of the control system showing how the acceleration sensor is used to position the electrohydraulic drive is shown in the graphic below (Figure 2). It consists of a simplified model of an electrohydraulic drive controlled by a PID controller, which generates a control signal for the actual drive, and a set-point compensation module based on the signal from an accelerometer mounted on the actuator’s piston rod. 

The first step was to home the drive by moving the actuator to its limit position by opening the valve for a few seconds. From that moment, it was possible to run the proposed algorithm (Figure 2).

**Figure 3:**
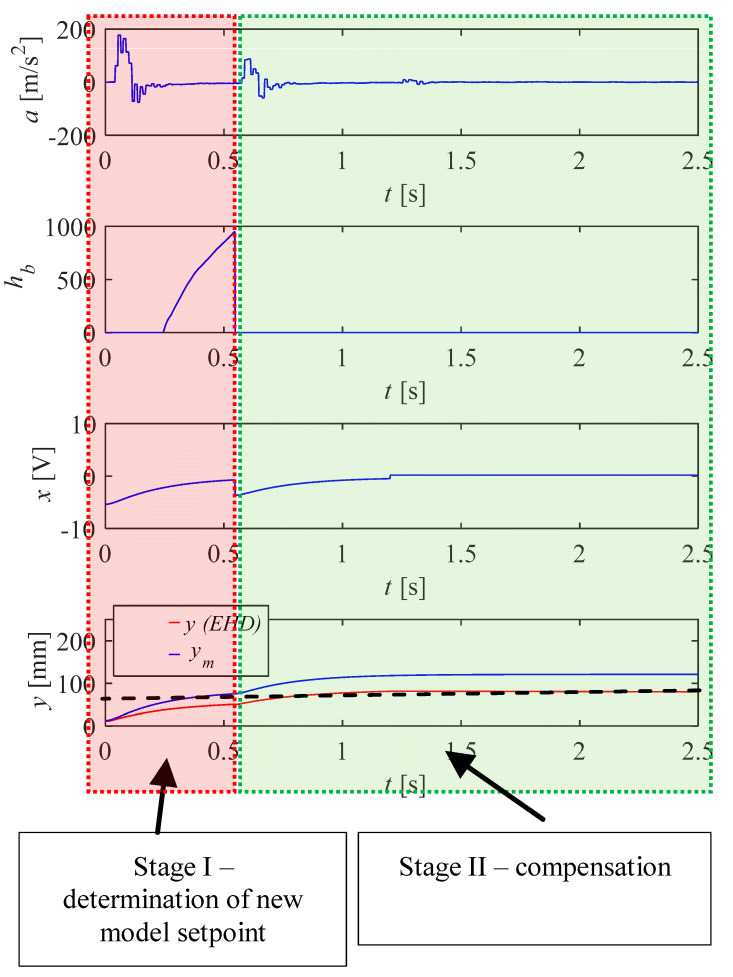
Graph showing the operation of the control algorithm, divided into two phases of movement.

Control of the electrohydraulic drive according to the method proposed in the article can be divided into two main phases (Figure 3):

- Phase I—Determination of a new set-point on the basis of the determined integral of the acceleration from the specified range of motion. This range begins when the position of the drive model reaches 70% of the reference value.
- Phase II—Compensation of the real drive position based on the signal from the model regulator.

The set value is determined on the basis of the following relationship. It depends on the current position of the drive:

(2) $$\{\begin{array}{cc} x=u & \mathrm{for}\;y_{m}\leq 0.8u\\ x=c_{a}\cdot c_{u}\cdot u & \mathrm{for}\;y_{m}>0.8u \end{array}$$

where *u*—set-point position; *x*—converted set value.

**Figure 4:**
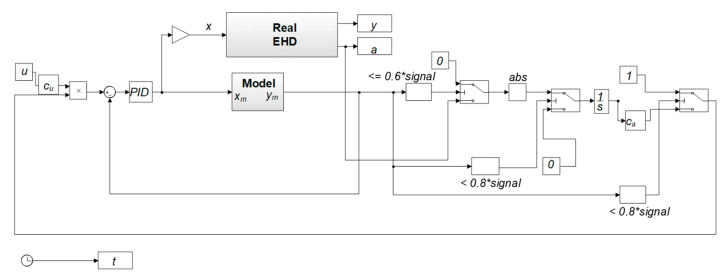
Simulation model of the drive and control system (EHD—electrohydraulic drive).

The detailed control scheme is presented in the diagram below (Figure 4). It is also a simulation model. It was prepared in the MATLAB Simulink environment. Furthermore, it has been divided into two main parts: the real electrohydraulic drive and a model of the control system including a simplified model. The simplified model was controlled by a PID controller. The signal from the regulator was sent to the input of the actual drive valve. The acceleration signal was compared with the position signal from the *y_m_* model.

**Figure 5:**
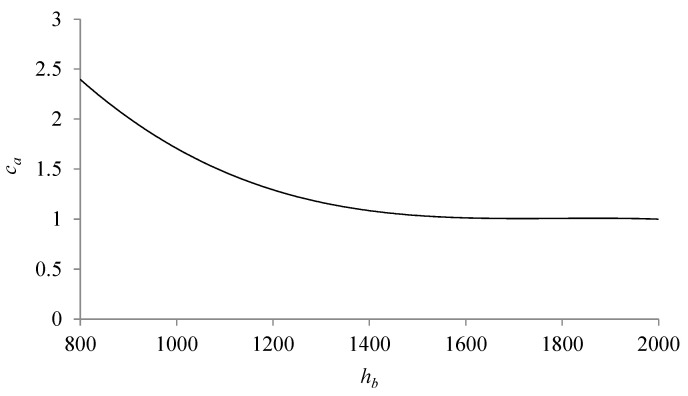
Correction function *c_a._*

In the motion range 0.6 < *y_m_* < 0.8, the integral of the acceleration signal from the real drive *y* was determined. The calculated value enters the *c_a_* curve. The curve takes into account the change in the calculated integral value depending on the change in external parameters (Figure 5), and in the case of the presented tests—pressure changes. The set signal *u* is additionally converted by means of the *c_u_* variable, the value of which was selected experimentally. The graph in Figure 5 was drawn on the basis of the data from Table 1 and Table 2. The average values of the *h_b_* parameter for individual pressure values were approximated by the polynomial described in Formula (3).

The corrective function *c_a_* for determining a new setpoint value is described by the following formula:

(3) $$c_{a}=-0.000000001h_{b}{}^{3}+0.00000001h_{b}{}^{2}-0.0139h_{b}+9.3046$$

The set-point-dependent correction function *c_u_* is described by the following formula:

(4) $$c_{u}=-0.0017u+1.47$$

The parameters of the above functions were selected experimentally.

## 3. Drive Model

In the first phase of movement, the control signal is determined, among others, based on a simplified model of a closed-loop electrohydraulic drive with a PID controller. This model did not have to include, for example, non-linearities such as non-linear friction characteristics (Stribeck model) or square root flow characteristics, because the value obtained in the second phase of motion was compensated Equation (3). Basic formulas describing electrohydraulic servo drives can be found in works by [25,26,27]. The simplified drive model was implemented on the basis of the following equations:

(5) $$m\frac{d^{2}y\left(t\right)}{dt^{2}}+F_{f}\left[v\left(t\right)\right]+F_{L}\left(t\right)=A\left[p_{a}\left(t\right)-ap_{b}\left(t\right)\right]$$

(6) $$F_{a}\left(t\right)=Ap_{a}\left(t\right)$$

(7) $$F_{b}\left(t\right)=aA\cdot p_{b}\left(t\right)$$

(8) $$\frac{d^{2}y\left(t\right)}{dt^{2}}=\frac{F_{L}\left(t\right)+F_{a}\left(t\right)-F_{b}\left(t\right)-F_{f}\left[v\left(t\right)\right]}{m}$$

(9) $$Q_{a}\left(t\right)=Q_{ha}\left(t\right)+Q_{sa}\left(t\right)$$

(10) $$Q_{b}\left(t\right)=Q_{hb}\left(t\right)+Q_{sb}\left(t\right)$$

(11) $$\frac{dp_{a}\left(t\right)}{dt}=\frac{E_{0}}{V_{a}}\cdot \left(Q_{a}\left(t\right)-A\frac{dy\left(t\right)}{dt}\right)$$

(12) $$\frac{dp_{b}\left(t\right)}{dt}=\frac{E_{0}}{V_{b}}\cdot \left(Q_{b}\left(t\right)-aA\frac{dy\left(t\right)}{dt}\right)$$

where *Q_a_*, *Q_b_*—flow to the cylinder chambers; *Q_ha_*, *Q_hb_*—absorption of the actuator chambers; *Q_sa_*, *Q_sb_*—flow of the covering losses due to compressibility; *p_a_*, *p_b_*—the pressure in the chambers of the actuator; *A*, *aA*—active surfaces of the piston rod; *V_a_*, *V_b_*—the volume of liquid in the chambers of the actuator; *F_f_*—friction force; *E_0_*—volume modulus of the oil; *m*—reduced mass loading.

The values of pressure and position were determined on both sides by integrating the equations using the numerical method of trapezoids.

The basic parameters of the tested model were as follows: *A* = 25 mm, *aA* = 40 mm and *E*_0_ = 1.2 × 10^9^ N/m^2^. In the case of the described method, the remaining parameters of the model do not have to be precisely defined due to the fact that the final set value is modified by the Equation (3). The model and other elements of the control system were implemented in the PLC controller using the Structured Text language.

## 4. Simulation Research

**Figure 6:**
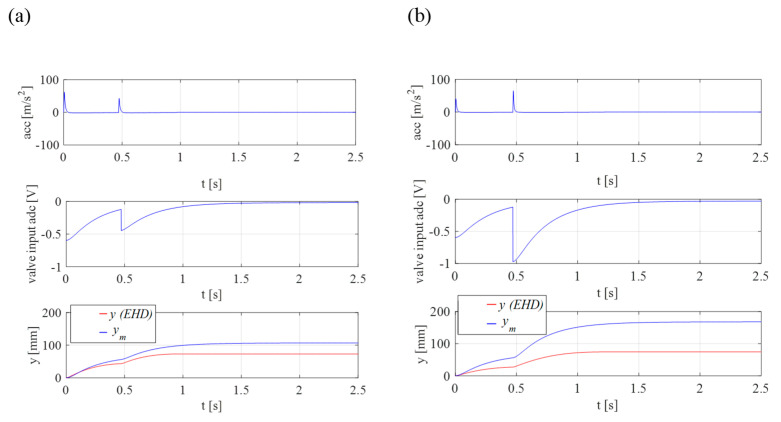
Simulation results for two values of supply pressure for the position *y* = 75 mm: (**a**) *p_0_* = 11 MPa; (**b**) *p_0_* = 5 MPa.

Before starting the experimental research, the author built a simulation model (Figure 4) and performed preliminary simulation tests. They were aimed at refining the control algorithm and checking the design assumptions. The graphs (Figure 6) show the simulation results for two selected values of the supply pressure *p_0_*: (a) 11 MPa and (b) 5 MPa. They show the influence of pressure variation compensation on the final position. The graphs show information about the displacement value for the reference model *y_m_*, the real drive *y*, the acceleration value *acc* and the control signal going to the drive. The performed tests were the starting point for further experimental research.

## 5. Experimental Test

**Figure 7:**
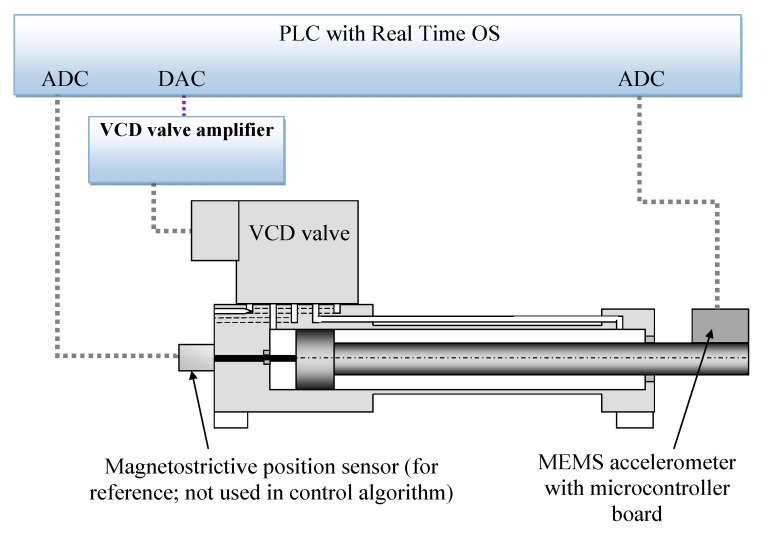
Control system diagram (test stand).

A diagram of the stand is presented in the diagram below (Figure 7). It consisted of a hydraulic actuator connected to a Voice Coil Drive proportional valve (Parker VCD [28]). The basic parameters of the valve were *Q* = 30 dm3/min, max. pressure *p_vmax_* = 30 MPa. The maximum stroke of the actuator was *y_max_* = 250 mm. The drive was powered from an external hydraulic power pack with the following parameters: max. power = 37 kW, maximum flow rate of 100 dm^3^/min and maximum pressure *p_0_max_* = 40 MPa. The drive was additionally equipped with a magnetostrictive sensor. It was not used in the control algorithm. It served only as a reference sensor for research purposes to know the actual position of the actuator (not to use in the control algorithm).

The main element of the control system (Figure 7) was a B&R Power Panel 500 PLC (5PP5X20) [29]. The controller was equipped with the following modules: an analog–digital ADC to which a reference magnetostrictive sensor was connected, an acceleration sensor with a microcontroller and a digital–analog converter (DAC) module which controlled the position of the valve spool. The controller was equipped with a 10-inch touch panel to facilitate testing. The controller ran under the control of the real-time operating system Automation Runtime, and the main control loop operated at *f* = 1 kHz.

**Figure 8:**
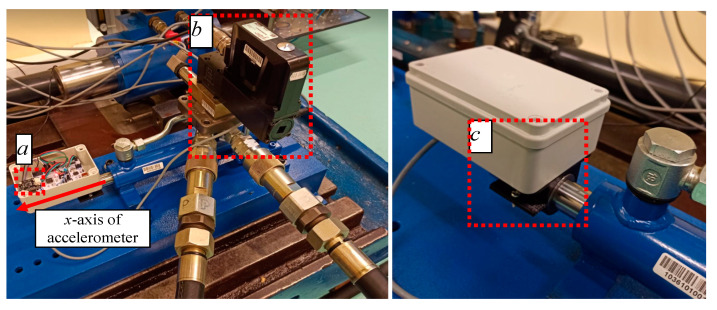
Construction of the test stand: (**a**) microelectromechanical system (MEMS) accelerometer connected with the STM32 microcontroller module, (**b**) VLC electrohydraulic valve and (**c**) sensor module cover with connector.

The project used a 32-bit STM32F432 microcontroller placed on the Nucleo development module (Figure 8a) [30]. A Bosch BNO055 MEMS inertial sensor [18] was connected to it via the I2C interface. This sensor consisted of an accelerometer, a gyroscope and a magnetometer. The basic parameters of the accelerometer were data reading in three independent axes; adjustable range of measured accelerations: ±2 g, ±4 g, ±8 g, ±16 g; resolution: 14 bits. The sensor was placed on the piston rod so that the linear movement was directed towards the *x*-axis (Figure 8). The accelerometer and the microcontroller were placed in a common housing. It was attached to the actuator with a screw connection (Figure 8c). The applied connection allows for tightening of subsequent elements to the piston rod. The read data were converted into an analog signal proportional to the acceleration, which went directly to the ADC inputs of the controller.

## 6. Results

**Figure 9:**
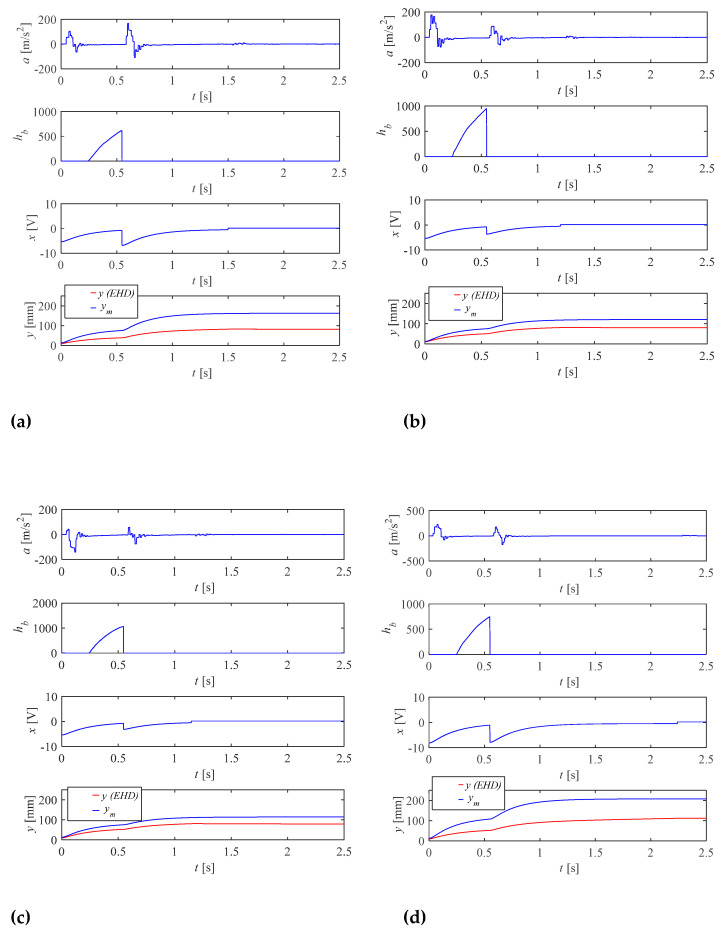

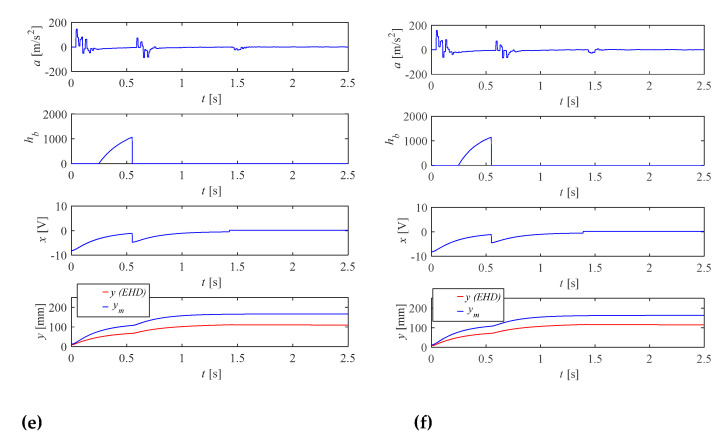
Acceleration *a*, calculated integral from acceleration *h_b_*, set-point hitting the drive valve *x* and actual position of the piston rod *y* for: (**a**) *p_0_* = 5 MPa, *u* = 80 mm; (**b**) *p_0_* = 9 MPa, *u* = 80 mm; (**c**) *p_0_* = 11 MPa, *u* = 80 mm; (**d**) *p_0_* = 5 MPa, *u* = 110 mm; (**e**) *p_0_* = 9 MPa, *u* = 110 mm; (**f**) *p_0_* = 11 MPa, *u* = 110 mm.

The experimental tests were carried out for two selected set-points: *u* = 80 mm and *u* = 110 mm (Figure 9). The pressure values were, respectively: 5, 9 and 11 MPa. The graphs show information about the displacement values for the reference model ym and the real drive y, the acceleration value of real drive *a* and the control signal going to the drive. The integral from acceleration *h_b_* used in the control system Equation (3) was calculated on the PLC. Pressure values for the hydraulic system were set as low as possible for safety reasons due to the prototype nature of the control system. The main purpose of the presented method was to also show the degree of effectiveness in the case of compensation in the flow of changes in the supply conditions of the hydraulic system.

**Figure 10:**
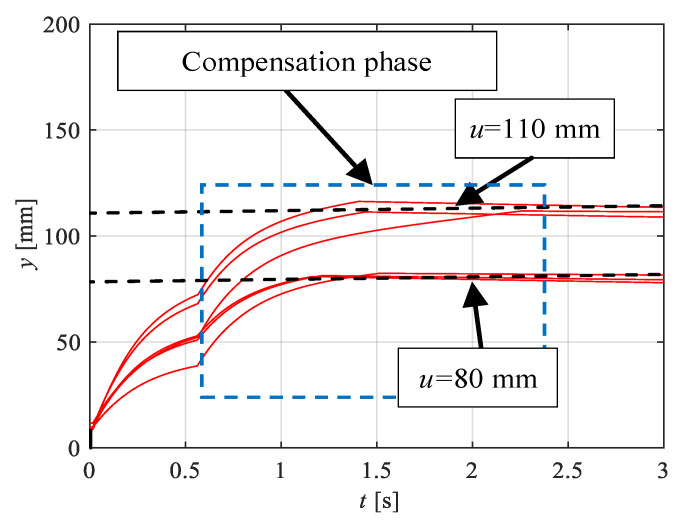
The electrohydraulic drive *y* position for the selected position values at the supply pressure *p_0_* of 5, 9 and 11 MPa (taken from the reference magnetostrictive sensor).

Figure 10 shows the positions of the electrohydraulic drive *y* for the previously indicated pressure values *p_0_*, collected in one diagram. The blue frame shows a part of the waveform, where the position value *y* is compensated based on the signal from the accelerometer *a* in accordance with the previously described algorithm.

Based on the tests performed, it can be concluded that the maximum position error was *e* = 4 mm. 

## 7. Discussion

The article presents simulation and experimental tests to verify the proposed control methods. The key problem was to find a way to read the data from the accelerometer in such a way that it would be possible to correctly determine the change in position of the actuator and, at the same time, ensure that measurement noise did not significantly affect the obtained measurement results. On the basis of the initial data collected, it was found that the data in the second phase of the movement should be analyzed when the accelerations are much lower. These conclusions result from the calculated value of the mean deviation in accordance with Table 1.

The acceleration values are integrated until the actuator reaches the specified position. On the basis of the obtained value, a compensation phase follows, where a new set value is determined. The aim of the research was to obtain a constant value of the position, regardless of the change of the supply pressure *p_0_*. Changing the supply pressure changes the acceleration of the system. Reducing this value leads to a decrease in the determined value of the acceleration integral.

Based on the research, it can be concluded that the best results can be achieved when the dynamics of actuator motion are limited. This can be done in two ways. One of them is limiting the supply pressure and pump flow. However, from a practical point of view it does not seem like a sensible solution. The second possibility is to limit the dynamics of the regulator by changing its selected parameters.

Based on the conducted research, it can be concluded that the proposed method has some disadvantages and limitations. In its present form, it is not suitable for positioning very precise drives. At the moment, the accelerometer module requires calibration each time it is placed on the new actuator. The author will focus on automating this process in his further works.

## 8. Conclusions

The aim of the research was to find a way to compensate for the disturbances occurring in a hydraulic cylinder using the data from a simplified model and an accelerometer mounted on the piston rod. The presented work showcases the compensation of pressure change influence on the positioning of an electrohydraulic drive. Tests were performed for three selected values of supply pressure.

From a practical point of view, the presented solution can be used in the case of already operating drives, where it is not possible to place a conventional position measuring system built into a hydraulic cylinder, such as a magnetostrictive sensor. The system described in the publication uses a sensor module with a microcontroller mounted directly on the piston rod by means of a threaded connection. Such a module can also be mounted in a different ways, e.g., by means of clamps. Since the control uses a signal coming from the range of motion with limited dynamics, the system can be configured for a specific application. The author wants to focus on this problem in further research.

At the same time, the proposed system can also act as a safety system, ensuring redundancy for the existing measuring system in the case of drives connected to very responsible systems (e.g., control of control systems used in aircraft). The proposed system can operate in parallel with a conventional position-measuring unit based on, e.g., a magnetostrictive sensor. Assembly of the module with the acceleration sensor does not require the removal of the hydraulic cylinder or its replacement.

To summarize, the main advantages of the solution proposed here are:

- The possibility to determine the position of the actuator and use it in the control system using data from the MEMS acceleration sensor;
- The relatively low price of the solution compared to magnetostrictive sensors and LVDT sensors mounted directly inside the actuator;
- The ease of assembly;
- The possibility to use a system to supervise the operation of the drive;
- The theoretical possibility of integration with various types of linear drives (e.g., electric drives).

In the next steps, the author plans to use the presented method to compensate for other disturbances, such as the influence of load. The ongoing work is also aimed at such a modification of the presented sensor so that it can work in a wireless mode. The author also plans to design a dedicated PCB with a microcontroller and acceleration sensor. The electronic system should be protected against unfavorable external conditions, e.g., hydraulic oil.

## Figures and Tables

**Table 1:** Selected test results for the parameter *h_a_*_,_ determined by mean deviation *σ*, arithmetic average, standard deviation estimator *s_x_* and measurement uncertainty *u*(*x*).

*p_0_* (MPa)	*h_a_* (Chosen Values to Illustrate the Problem)	Mean Deviation *σ*	Arithmetic Average	*s_x_*	*u*(*x*)
11	6835	1390.75	4505.40	1753.96	554.65
11	2813				
11	4441				
9	3202	1469.28	4493.20	1701.00	537.90
9	6962				
9	3527				
5	3168	366.92	3334.80	430.83	136.24
5	3977				
5	2987				

**Table 2:** Selected test results for the parameter *h_b_*_,_ determined by mean deviation *σ*, arithmetic average, standard deviation estimator *s_x_* and measurement uncertainty *u*(*x*).

*p_0_* (MPa)	*h_b_* (Chosen Values to Illustrate the Problem)	Mean Deviation *σ*	Arithmetic Average	*s_x_*	*u*(*x*)
11	1123	7.70	1127.70	9.51	3.01
11	1118				
11	1137				
9	983	7.30	979.10	8.89	2.81
9	967				
9	990				
5	618	23.92	662.40	29.58	9.35
5	683				
5	699				

## Data Availability

Not applicable.
